# When self-compassion matters: romantic relationship quality and infidelity proneness

**DOI:** 10.3389/fpsyg.2026.1945800

**Published:** 2026-10-01

**Authors:** Hatice Deniz Özdemir, Tubanur Bayram Kuzgun

**Affiliations:** 1 School of Arts and Social Sciences, Psychology, Esenyurt University, İstanbul, Türkiye; 2 Department of Psychology, Cappadocia University, Nevsehir, Türkiye; 3 Ghost to Guest Institute, Istanbul, Türkiye

**Keywords:** couple psychotherapy, infidelity proneness, moderation, romantic relationship quality, romantic relationships, self-compassion

## Abstract

Infidelity proneness reflects an individual’s tendency or inclination toward infidelity and is conceptually distinct from enacted infidelity. Although lower romantic relationship quality has been associated with greater infidelity-related intentions, less is known about the intrapersonal characteristics that may condition this association. The present study examined whether self-compassion moderates the association between perceived romantic relationship quality and infidelity proneness. Using a cross-sectional correlational design, data were collected from 384 adults in Türkiye aged 18–68 years (*M* = 31.04, *SD* = 9.40). Participants completed measures of perceived romantic relationship quality, self-compassion, and infidelity proneness. Moderation analysis was conducted using PROCESS Model 1, adjusting for gender, age, and relationship duration. Perceived romantic relationship quality was negatively associated with infidelity proneness (*r* = −0.614, *p <* 0.001), as was self-compassion (*r* = −0.251, *p* < 0.001). Self-compassion significantly moderated the association between perceived romantic relationship quality and infidelity proneness (*b* = 0.062, SE = 0.015, *p* < 0.001), accounting for an additional 2.2% of the variance. The negative association between relationship quality and infidelity proneness was significant at low, mean, and high levels of self-compassion but became progressively weaker as self-compassion increased. At lower levels of relationship quality, higher self-compassion corresponded to lower predicted infidelity proneness; however, this pattern was not uniform across the full range of relationship quality. These findings suggest that self-compassion may be relevant to understanding individual differences in how relational evaluations are associated with infidelity-related tendencies, while cautioning against interpreting self-compassion as uniformly protective. From a clinical perspective, the findings support considering both perceptions of relationship quality and intrapersonal regulation when assessing infidelity-related concerns in individual and couple contexts, while longitudinal research is needed before causal or intervention-related conclusions can be drawn.

## 1 Introduction

Infidelity represents a consequential challenge in romantic relationships, with implications for both relational stability and individual well-being. Enacted infidelity, commonly referring to emotional and/or sexual involvement outside the boundaries agreed upon within a committed relationship, has been associated with adverse relational and psychological outcomes (Blow and Hartnett, 2005; Rokach and Chan, 2023). Among individuals who have experienced a partner’s infidelity, research has documented associations with relationship dissolution and depressive outcomes (Hall and Fincham, 2006; Whisman, 2016).

A distinction is therefore necessary between enacted infidelity and constructs reflecting an inclination toward infidelity. Whereas enacted infidelity refers to behavior that violates the agreed-upon exclusivity of a romantic relationship, constructs such as infidelity intentions, propensity, and proneness concern individuals’ willingness, inclination, or susceptibility to engage in such behavior. These constructs should not be treated as equivalent to actual infidelity, nor does the presence of such tendencies imply that infidelity will occur. Rather, they permit the examination of individual differences in vulnerability to infidelity without conflating psychological inclination with enacted behavior. Research has accordingly examined infidelity intentions as an outcome in its own right, including among individuals in exclusive romantic relationships and in Turkish samples (Toplu-Demirtaş and Tezer, 2013; Urganci et al., 2021; Selalmaz and Erdem, 2025). In the present study, infidelity proneness refers specifically to an individual’s tendency or inclination toward infidelity rather than the occurrence of enacted infidelity.

Perceived romantic relationship quality represents one relational factor that may be particularly relevant to individual differences in infidelity proneness. Relationship quality reflects individuals’ broader evaluations of their romantic relationship, encompassing satisfaction, commitment, intimacy, trust, passion, and love (Fletcher et al., 2000). Lower relationship quality may be accompanied by unmet emotional or relational needs and reduced investment in the primary relationship, conditions that have been associated with infidelity-related outcomes (Haseli et al., 2019; Rokach and Chan, 2023). Importantly, evidence also links evaluations of the primary relationship with intentions toward infidelity, rather than only with enacted behavior. Among individuals in exclusive romantic relationships, poorer relationship quality has been associated with greater intentions toward infidelity (Urganci et al., 2021). Although intentions and proneness are not interchangeable constructs, this evidence suggests that less favorable evaluations of one’s romantic relationship may be associated with greater psychological inclination toward infidelity. Perceived romantic relationship quality therefore provides an important relational context within which infidelity proneness can be examined.

Nevertheless, perceived romantic relationship quality alone may be insufficient to account for individual differences in infidelity proneness. Individuals experiencing comparable levels of relational difficulty may differ considerably in how they interpret, regulate, and respond to those experiences. Research on infidelity-related outcomes likewise points to the relevance of both relational and individual characteristics rather than a single determinant (Haseli et al., 2019; Urganci et al., 2021). From this perspective, the association between perceived romantic relationship quality and infidelity proneness may depend partly on the intrapersonal resources individuals bring to relational experiences. One such resource is self-compassion.

Originally conceptualized by Neff (2003a,b), self-compassion involves responding to personal difficulty with self-kindness rather than harsh self-judgment, recognizing distress as part of a shared human experience rather than as an isolating failure, and maintaining mindful awareness rather than becoming overidentified with painful thoughts and emotions. Self-compassion therefore concerns how individuals relate to themselves when confronting distress rather than the absence of distress itself. Importantly for the present model, previous research has examined self-compassion not only as a correlate of psychological functioning but also as a moderator of associations between stressful experiences and psychological outcomes (Dev et al., 2020). This provides a conceptual basis for considering whether individuals with different levels of self-compassion may respond differently to relational difficulties.

This regulatory function may be particularly relevant in romantic relationships, where dissatisfaction, conflict, rejection, or unmet relational needs can evoke distress and require regulation of both self-directed and interpersonal responses. Because self-compassion is characterized by less self-criticism and overidentification in response to distress, individuals higher in self-compassion may be less reactive to unfavorable relationship experiences, potentially weakening the extent to which lower perceived relationship quality is associated with infidelity proneness. Existing evidence indicates that self-compassion is associated with adaptive functioning in close relationships. Higher self-compassion has been associated with a greater likelihood of compromise, lower emotional turmoil during interpersonal conflict, and greater relational well-being, including in conflicts involving romantic partners (Yarnell and Neff, 2013). Self-compassion has also been associated with more constructive romantic relationship behavior (Neff and Beretvas, 2013). More recent dyadic research indicates that both general and relationship-specific self-compassion are positively associated with individuals’ own relationship satisfaction, whereas relationship-specific self-compassion is also associated with partners’ relationship satisfaction (Körner et al., 2024). Consistent with these findings, a scoping review of 72 studies concluded that self-compassion is broadly associated with thoughts, emotions, and behaviors occurring within close interpersonal relationships, including healthier romantic functioning and more constructive responses to conflict and relational transgressions (Lathren et al., 2021).

Together, this literature suggests that self-compassion may be relevant not only to individuals’ evaluations of their relationships but also to how they respond when relational experiences become difficult. Applied to infidelity proneness, self-compassion may therefore represent an intrapersonal characteristic that alters the extent to which tendencies toward infidelity are associated with perceived romantic relationship quality. Importantly, conceptualizing self-compassion as a moderator does not require the temporal assumption that relationship quality first produces changes in self-compassion. Rather, it tests whether the association between perceived romantic relationship quality and infidelity proneness differs across levels of self-compassion.

### 1.1 The present study

Although perceived romantic relationship quality has been associated with infidelity-related intentions, less is known about intrapersonal characteristics that may alter the strength of this association. Existing research has examined self-compassion in relation to psychological adjustment and romantic relationship functioning, but its potential moderating role in the association between perceived romantic relationship quality and infidelity proneness remains underexplored. Addressing this gap is important because individuals reporting similar levels of relationship quality may differ in the extent to which their evaluations of the relationship are associated with tendencies toward infidelity.

Accordingly, the present study examined the associations among perceived romantic relationship quality, self-compassion, and infidelity proneness and tested whether self-compassion moderated the association between perceived romantic relationship quality and infidelity proneness. We hypothesized that higher perceived romantic relationship quality would be associated with lower infidelity proneness (H1). We further hypothesized that higher self-compassion would be associated with lower infidelity proneness (H2). Finally, we hypothesized that self-compassion would moderate the association between perceived romantic relationship quality and infidelity proneness, such that the strength of this association would vary across levels of self-compassion (H3). The hypothesized moderation model is presented in Figure 1.

**FIGURE 1:**
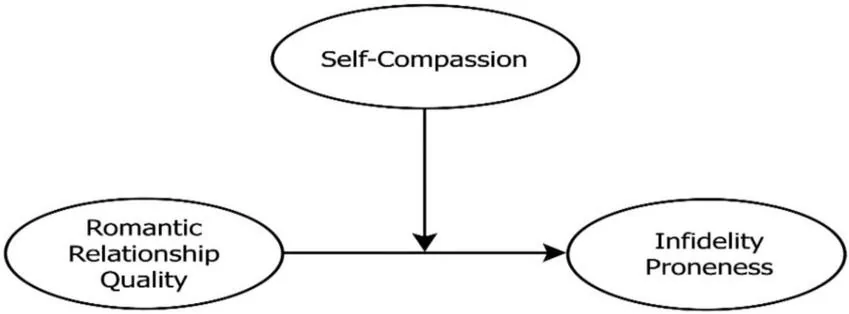
Hypothesized moderating role of self-compassion in the association between perceived romantic relationship quality and infidelity proneness.

## 2 Materials and methods

### 2.1 Study design

This cross-sectional, correlational study examined whether self-compassion moderated the association between perceived romantic relationship quality and infidelity proneness. Perceived romantic relationship quality was specified as the focal predictor, infidelity proneness as the outcome variable, and self-compassion as the moderator. Gender, age, and relationship duration were included as covariates in the moderation model.

### 2.2 Participants and procedure

Participants were adults aged 18 years or older who resided in Türkiye and were either currently involved in a romantic relationship or had been involved in one within the previous year. Eligible relationship experiences included marriage, engagement, dating, or cohabitation. Participation was voluntary, and all participants provided informed consent before completing the study measures.

Data were collected online via Google Forms. A total of 385 participants initially completed the survey. During data screening, one response containing missing data was excluded, resulting in a final analytic sample of 384 participants.

Participants ranged in age from 18 to 68 years (*M* = 31.04, *SD* = 9.40). Of the final sample, 269 participants (70.1%) were women and 115 (29.9%) were men. Regarding educational level, 296 participants (77.1%) were enrolled in or had completed an undergraduate degree, whereas 88 (22.9%) had postgraduate-level education. In terms of self-perceived socioeconomic status, 188 participants (49.0%) described their socioeconomic status as moderate, 172 (44.8%) as good, 12 (3.1%) as very good, and 12 (3.1%) as poor.

Relationship duration was distributed as follows: 51 participants (13.3%) reported a relationship lasting 1–12 months, 142 (37.0%) reported 1–3 years, 92 (24.0%) reported 3–10 years, and 99 (25.8%) reported a relationship duration of 10 years or longer.

Ethical approval was obtained from the Biruni University Non-Interventional Research Ethics Committee (21 October 2024; Decision No. 2024 BIAEK/04-29). All participants provided informed consent before participation.

### 2.3 Measures

#### 2.3.1 Perceived romantic relationship quality scale

Perceived romantic relationship quality was assessed using the Perceived Romantic Relationship Quality Scale (PRRQS), originally developed by Fletcher et al. (2000) and adapted into Turkish by Sağkal and Özdemir (2018). The scale consists of six items rated on a 7-point Likert-type response format. The Turkish adaptation demonstrated good internal consistency (Cronbach’s α = 0.86). In the present study, internal consistency was high (Cronbach’s α = 0.89).

#### 2.3.2 Infidelity proneness scale

Infidelity proneness was assessed using the Infidelity Proneness Scale developed by Polat (2006). The scale consists of 30 items rated on a 5-point Likert-type response format, with higher scores indicating greater proneness toward infidelity. Twelve items are reverse scored (Items 4, 9, 10, 11, 14, 16, 17, 18, 20, 23, 27, and 28). In the original scale-development study, internal consistency was high (Cronbach’s α = 0.95). In the present sample, Cronbach’s α was 0.94.

#### 2.3.3 Self-compassion scale–short form

Self-compassion was assessed using the 12-item Self-Compassion Scale–Short Form (SCS-SF), originally developed by Raes et al. (2011) and adapted into Turkish for adults by Özdemir and Deniz (2025). Items are rated on a 5-point Likert-type scale, with Items 1, 4, 8, 9, 11, and 12 reverse scored when calculating the total self-compassion score. The Turkish version demonstrated good internal consistency (Cronbach’s α = 0.86) and high test–retest reliability (r = 0.94; Özdemir and Deniz, 2025). In the present sample, Cronbach’s α was 0.80.

### 2.4 Data analysis

Statistical analyses were conducted using IBM SPSS Statistics version 22 and the PROCESS macro developed by Hayes. Prior to the primary analyses, the distributions of the main study variables were examined using skewness and kurtosis coefficients together with the Kolmogorov–Smirnov test. Because significance tests of normality can be sensitive to relatively minor deviations in larger samples, distributional characteristics were evaluated primarily on the basis of skewness and kurtosis values. Descriptive statistics, including means and standard deviations, were calculated for the main study variables.

Bivariate associations among perceived romantic relationship quality, self-compassion, and infidelity proneness were examined using Pearson product–moment correlations. Spearman rank-order correlations were additionally calculated as a robustness check for possible departures from normality.

To examine whether self-compassion moderated the association between perceived romantic relationship quality and infidelity proneness, a moderation analysis was conducted using PROCESS Model 1 (Hayes, 2022). Perceived romantic relationship quality was entered as the focal predictor, self-compassion as the moderator, and infidelity proneness as the outcome variable. Gender, age, and relationship duration were included as covariates.

Because relationship duration was assessed using four categories with unequal time intervals (1–12 months, 1–3 years, 3–10 years, and 10 years or longer), it was represented in the regression model using three dummy-coded variables. The 1–12-month category served as the reference group; separate dummy variables represented relationships lasting 1–3 years, 3–10 years, and 10 years or longer. Gender was coded as 1 = women and 2 = men.

Moderation was evaluated using the interaction term between perceived romantic relationship quality and self-compassion. When the interaction term was statistically significant, conditional associations between perceived romantic relationship quality and infidelity proneness were estimated at low (-1 *SD*), mean, and high (+1 *SD*) levels of self-compassion. Statistical significance was evaluated at α = 0.05, and 95% confidence intervals were reported for parameter estimates.

### 2.5 Sample size and power analysis

An a priori power analysis was conducted using G*Power 3.1.9.7 to determine the minimum sample size required to detect the interaction term in the moderation model. The analysis was specified as a linear multiple regression fixed model testing the increase in explained variance attributable to the interaction term. Assuming an effect size of *f*^2^ = 0.05, α = 0.05, statistical power of 0.95, one tested predictor corresponding to the interaction term, and eight total predictors in the regression model, the estimated minimum required sample size was *N* = 262. The final analytic sample of 384 participants therefore exceeded this threshold.

## 3 Results

### 3.1 Descriptive statistics and preliminary analyses

Descriptive statistics and internal consistency coefficients for the study variables are presented in Table 1. The mean scores were 34.25 (SD = 7.58) for perceived romantic relationship quality, 38.18 (SD = 7.60) for self-compassion, and 57.13 (SD = 20.97) for infidelity proneness. Cronbach’s alpha coefficients were 0.89, 0.80, and 0.94, respectively.

**TABLE 1:** Descriptive statistics and internal consistency coefficients for the study variables.

Variable	*M*	*SD*	Skewness	Kurtosis	Cronbach’s α
Perceived romantic relationship quality	34.25	7.58	−1.309	1.357	0.89
Self-compassion	38.18	7.60	−0.166	0.001	0.80
Infidelity proneness	57.13	20.97	1.340	2.715	0.94

*N* = 384.

Skewness values ranged from −1.31 to 1.34, and kurtosis values ranged from 0.001 to 2.72. Although the Kolmogorov–Smirnov tests were statistically significant for all three variables, inspection of the skewness and kurtosis coefficients did not indicate substantial univariate distributional problems.

### 3.2 Bivariate associations

Pearson correlations among perceived romantic relationship quality, self-compassion, and infidelity proneness are presented in Table 2. Perceived romantic relationship quality was negatively associated with infidelity proneness, *r* = −0.614, *p <* 0.001, and positively associated with self-compassion, *r* = 0.253, *p <* 0.001. Self-compassion was also negatively associated with infidelity proneness, *r* = −0.251, *p <* 0.001.

**TABLE 2:** Pearson correlations among the study variables.

Variable	1	2	3
1. Perceived romantic relationship quality	–	–	–
2. Infidelity proneness	−0.614***		
3. Self-compassion	0.253***	−0.251***	

***p < 0.001.

As a robustness check for potential deviations from normality, the correlations were re-examined using Spearman rank-order correlations. The directions and statistical significance of the associations remained unchanged.

### 3.3 Moderation analysis

A moderation analysis was conducted using PROCESS Model 1 to examine whether self-compassion moderated the association between perceived romantic relationship quality and infidelity proneness, adjusting for gender, age, and relationship duration. Relationship duration was represented by three dummy-coded variables, with relationships lasting 1–12 months serving as the reference category.

The overall regression model was statistically significant, *F*(8, 375) = 47.679, *p <* 0.001, and accounted for 50.4% of the variance in infidelity proneness (*R* = 0.710, *R*^2^ = 0.504; Table 3). The interaction between perceived romantic relationship quality and self-compassion was statistically significant, *b* = 0.062, SE = 0.015, *t* = 4.112, *p <* 0.001, 95% CI [0.032, 0.091]. The interaction term accounted for an additional 2.2% of the variance in infidelity proneness, Δ*R*^2^ = 0.022, *F*(1, 375) = 16.906, *p <* 0.001. Thus, the association between perceived romantic relationship quality and infidelity proneness varied as a function of self-compassion.

**TABLE 3:** Moderation model predicting infidelity proneness.

Predictor	*b*	*SE*	*t*	*p*	95% CI
Constant	187.649	20.157	9.309	<0.001	[148.014, 227.284]
Perceived romantic relationship quality	−3.830	0.552	−6.943	<0.001	[−4.915, −2.745]
Self-compassion	−2.398	0.535	−4.479	<0.001	[−3.451, −1.346]
Relationship quality × Self-compassion	0.062	0.015	4.112	<0.001	[0.032, 0.091]
Gender	10.465	1.740	6.015	<0.001	[7.044, 13.887]
Relationship duration: 1–3 years	6.530	2.458	2.656	0.008	[1.697, 11.363]
Relationship duration: 3–10 years	8.528	2.729	3.124	0.002	[3.161, 13.895]
Relationship duration: ≥10 years	6.614	3.384	1.954	0.051	[−0.040, 13.269]
Age	−0.301	0.122	−2.472	0.014	[−0.541, −0.062]

*N* = 384. *R* = 0.710, *R*^2^ = .504, MSE = 222.618, *F*(8, 375) = 47.679, *p <* 0.001. The interaction term accounted for Δ*R*^2^ = 0.022, *F*(1, 375) = 16.906, *p <* 0.001. Gender was coded 1 = women and 2 = men. Relationship duration was dummy coded with 1–12 months as the reference category.

Among the covariates, gender was significantly associated with infidelity proneness, *b* = 10.465, SE = 1.740, *t* = 6.015, *p <* 0.001, 95% CI [7.044, 13.887]. Given the coding of gender (1 = women, 2 = men), men reported higher infidelity proneness scores than women, conditional on the other variables included in the model. Age was negatively associated with infidelity proneness, *b* = −0.301, SE = 0.122, *t* = −2.472, *p* = 0.014, 95% CI [−0.541, −0.062].

With respect to relationship duration, participants reporting relationships of 1–3 years had higher infidelity proneness scores than participants in the 1–12-month reference group, *b* = 6.530, SE = 2.458, *t* = 2.656, *p* = 0.008, 95% CI [1.697, 11.363]. Participants reporting relationships of 3–10 years likewise had higher infidelity proneness scores than the reference group, *b* = 8.528, SE = 2.729, *t* = 3.124, *p* = 0.002, 95% CI [3.161, 13.895]. The difference between participants reporting relationships of 10 years or longer and those in the 1–12-month reference group did not reach statistical significance at the 0.05 level, *b* = 6.614, SE = 3.384, *t* = 1.954, *p* = 0.051, 95% CI [−0.040, 13.269].

Because the interaction was statistically significant, the conditional associations between perceived romantic relationship quality and infidelity proneness were examined at low (-1 SD), mean, and high (+1 SD) levels of self-compassion (Table 4). Perceived romantic relationship quality was negatively associated with infidelity proneness at all three levels of self-compassion.

**TABLE 4:** Conditional associations between perceived romantic relationship quality and infidelity proneness at different levels of self-compassion.

Self-compassion	Value	*b*	*SE*	*t*	*p*	95% CI
Low (-1 *SD*)	30.577	−1.937	0.133	−14.513	<0.001	[−2.200, −1.675]
Mean	38.185	−1.466	0.111	−13.188	<0.001	[−1.685, −1.248]
High (+1 *SD*)	45.792	−0.996	0.182	−5.469	<0.001	[−1.354, −0.638]

Conditional associations were estimated at the mean and one standard deviation below and above the mean of self-compassion. PROCESS explicitly identifies these values as the mean and ± 1 SD.

At low self-compassion (*W* = 30.577), the conditional association was *b* = −1.937, SE = 0.133, *t* = −14.513, *p <* 0.001, 95% CI [−2.200, −1.675]. At the mean level of self-compassion (*W* = 38.185), the conditional association was *b* = −1.466, *SE* = 0.111, *t* = −13.188, *p <* 0.001, 95% CI [−1.685, −1.248]. At high self-compassion (*W* = 45.792), the conditional association remained statistically significant but was smaller in magnitude, *b* = −0.996, *SE* = 0.182, *t* = −5.469, *p <* 0.001, 95% CI [−1.354, −0.638].

As illustrated in Figure 2, the negative association between perceived romantic relationship quality and infidelity proneness became progressively weaker as self-compassion increased. At lower levels of perceived romantic relationship quality, higher self-compassion was associated with lower predicted infidelity proneness. This pattern was consistent with the statistically significant interaction and indicated that the magnitude of the association between perceived romantic relationship quality and infidelity proneness varied across levels of self-compassion.

**FIGURE 2:**
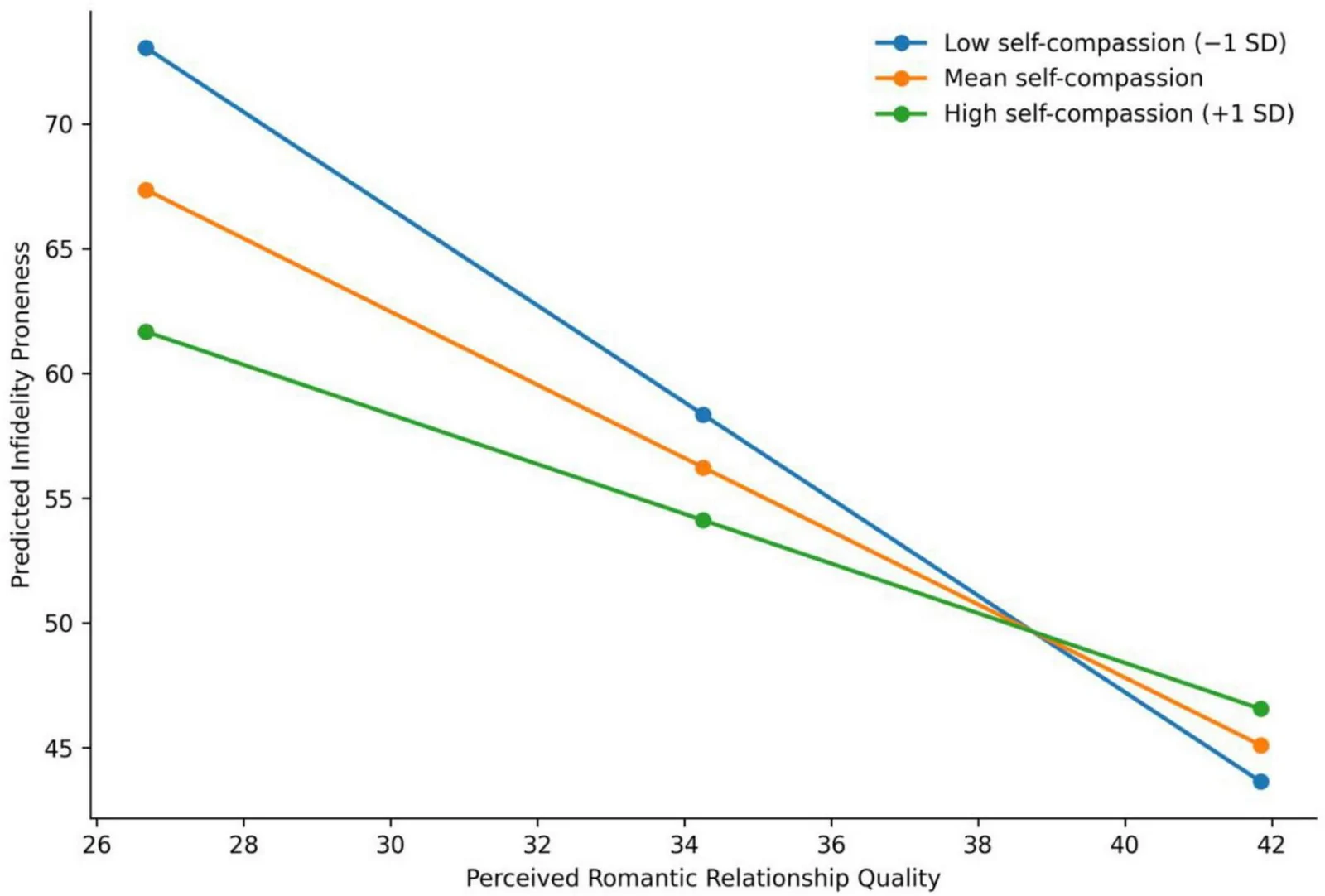
Association between perceived romantic relationship quality and infidelity proneness at low, mean, and high levels of self-compassion. Low, mean, and high self-compassion correspond to one standard deviation below the mean, the mean, and one standard deviation above the mean, respectively.

## 4 Discussion

The present study examined the associations among perceived romantic relationship quality, self-compassion, and infidelity proneness and tested whether self-compassion moderated the association between perceived romantic relationship quality and infidelity proneness. Three main findings emerged. First, higher perceived romantic relationship quality was associated with lower infidelity proneness. Second, higher self-compassion was also associated with lower infidelity proneness. Third, and most importantly, self-compassion significantly moderated the association between perceived romantic relationship quality and infidelity proneness after accounting for gender, age, and relationship duration. Although perceived romantic relationship quality was negatively associated with infidelity proneness at all examined levels of self-compassion, the magnitude of this negative association became progressively weaker as self-compassion increased. Together, these findings suggest that infidelity proneness is associated not only with individuals’ evaluations of their romantic relationships but also with intrapersonal characteristics that may condition the strength of this association.

Consistent with H1, perceived romantic relationship quality was negatively associated with infidelity proneness. This finding accords with previous evidence indicating that less favorable evaluations of a primary relationship are associated with greater intentions toward infidelity. In particular, Urganci et al. (2021) reported that poorer relationship quality was associated with stronger infidelity intentions among individuals in exclusive romantic relationships. Although infidelity intentions and infidelity proneness are conceptually distinct and should not be equated with enacted infidelity, the present findings extend this general pattern to individuals’ broader inclination toward infidelity. Rather than implying that dissatisfaction inevitably leads to infidelity, the findings indicate that individuals who evaluate their romantic relationships more positively tend to report lower proneness toward infidelity.

Consistent with H2, self-compassion was negatively associated with infidelity proneness. This finding is compatible with a broader literature linking self-compassion with more adaptive functioning in close relationships. Previous research has associated self-compassion with more constructive responses to interpersonal conflict and greater relational well-being, whereas dyadic research has linked both general and relationship-specific self-compassion with relationship satisfaction (Yarnell and Neff, 2013; Körner et al., 2024). A broader review similarly indicates that self-compassion is relevant to how individuals think, feel, and behave within close relationships (Lathren et al., 2021). The present findings extend this literature by suggesting that self-compassion is also negatively associated with infidelity proneness, an outcome that has received comparatively limited attention in the self-compassion literature.

The central finding concerned H3. Self-compassion significantly moderated the association between perceived romantic relationship quality and infidelity proneness. The interaction accounted for an additional 2.2% of the variance in infidelity proneness beyond the other terms in the model, Δ*R*^2^ = 0.022, *F*(1, 375) = 16.906, *p <* 0.001. Examination of the conditional associations showed a systematic pattern. The negative association between perceived romantic relationship quality and infidelity proneness was strongest at low self-compassion (*b* = −1.937), weaker at the mean level (*b* = −1.466), and weakest, although still statistically significant, at high self-compassion (*b* = −0.996). Thus, H3 was supported in the hypothesized sense that the strength of the relationship quality–infidelity proneness association varied across levels of self-compassion.

This interaction warrants a more nuanced interpretation than a simple buffering account. The predicted values indicate that, at lower levels of perceived romantic relationship quality, higher self-compassion corresponded to lower predicted infidelity proneness. However, as perceived romantic relationship quality increased, the predicted values across self-compassion levels converged and changed ordering toward the upper end of relationship quality. Accordingly, the present interaction should not be interpreted as evidence that self-compassion uniformly protects against infidelity proneness across the full range of relationship quality.

One possible interpretation is that self-compassion may reduce the extent to which infidelity-related tendencies covary with negative evaluations of the current relationship. Self-compassion involves responding to distress with less self-criticism and overidentification and has been associated with more adaptive responses to interpersonal difficulty. Previous work has also suggested that the relational implications of self-compassion may depend on other personal and motivational characteristics, rather than being uniformly beneficial across all relational contexts (Baker and McNulty, 2011). From this perspective, individuals higher in self-compassion may be less reactive to unfavorable evaluations of their romantic relationships, such that lower perceived relationship quality is less strongly associated with infidelity proneness. However, the present cross-sectional data do not identify the psychological process responsible for the observed interaction. This interpretation should therefore be regarded as a theoretical possibility rather than as evidence of a demonstrated regulatory mechanism.

The crossing pattern also raises an important question for future research. At higher levels of perceived romantic relationship quality, greater self-compassion did not correspond to the lowest predicted infidelity proneness. This finding cautions against conceptualizing self-compassion as a straightforward inhibitor of infidelity-related tendencies. Self-compassion may facilitate emotional regulation and reduce distress without necessarily increasing commitment to a particular relationship or strengthening motivation to preserve that relationship under all circumstances. Future research could therefore examine whether commitment, attachment orientation, relationship-specific self-compassion, relationship maintenance motivation, or emotion-regulation processes help explain when self-compassion is associated with lower versus higher infidelity proneness.

The covariates also revealed several noteworthy associations. Men reported higher infidelity proneness than women after adjustment for the other variables in the model, *b* = 10.465, *p <* 0.001. Age was negatively associated with infidelity proneness, *b* = −0.301, *p* = 0.014. Relationship duration showed a non-monotonic pattern. Relative to participants reporting relationships of 1–12 months, those reporting relationships of 1–3 years and 3–10 years had higher infidelity proneness scores, whereas the difference for relationships of 10 years or longer did not reach conventional statistical significance. This non-monotonic pattern indicates that relationship duration should not be interpreted as showing a simple linear increase in infidelity proneness. Because relationship duration was included primarily as a covariate rather than as a focal predictor, these differences should be interpreted cautiously and examined directly in future research.

### 4.1 Cultural context

The Turkish sociocultural context warrants specific consideration because relationship evaluations and infidelity-related tendencies may be embedded within broader norms concerning marriage, family relationships, gender, sexuality, and relational exclusivity. Previous research on dating infidelity has situated Türkiye as a relatively collectivistic and predominantly Muslim cultural context and emphasized the importance of considering culturally specific aspects of infidelity within this setting (Toplu-Demirtaş and Fincham, 2018). Research on urban Turkish couples further suggests that marital relationships may be intertwined with relationships with families of origin. In a study of 430 Turkish couples across different stages of the marital life cycle, Hortaçsu (2007) found that, compared with family-initiated marriages, couple-initiated marriages were less enmeshed with families of origin, more egalitarian, and more emotionally involving. These findings illustrate the potential relevance of broader family relational networks to marital functioning while also indicating heterogeneity in relationship patterns within Türkiye.

Gendered norms concerning sexuality and infidelity may be particularly relevant to the present findings. In a sample of 420 Turkish university students, men reported more favorable attitudes toward infidelity and stronger intentions toward infidelity than women (Toplu-Demirtaş and Fincham, 2018). This pattern parallels the present finding that men reported higher infidelity proneness than women after adjustment for age, relationship duration, perceived relationship quality, self-compassion, and the interaction term. Evidence from Türkiye also indicates that attitudes toward sexual behavior are associated with traditional gender-role beliefs and religiosity. Using data from the 2011 World Values Survey in Türkiye (N = 1,605), Karaman et al. (2022) found that traditional gender-role beliefs and indicators of religiosity were associated with more negative attitudes toward premarital sex, with traditional gender roles emerging as a particularly strong correlate of conservative sexual attitudes. Earlier research similarly found that benevolent sexism uniquely predicted more negative evaluations of women who engaged in premarital sex among Turkish participants after other relevant variables were controlled (Sakallı-Uğurlu and Glick, 2003). Although the latter studies did not examine infidelity specifically, together these findings suggest that sexuality and relationship-boundary behaviors in Türkiye may be evaluated within gendered and normative frameworks. This is relevant to the present study because infidelity proneness is a socially sensitive self-report construct, and endorsement of infidelity-related tendencies may partly reflect the normative context in which such tendencies are evaluated.

These sociocultural considerations may also provide a context for interpreting the observed moderation pattern, although they cannot establish a culturally specific explanation for it. At lower levels of perceived romantic relationship quality, participants higher in self-compassion showed lower predicted infidelity proneness, whereas differences across levels of self-compassion converged as perceived relationship quality increased. One possibility is that self-compassion may become particularly relevant when individuals are experiencing unfavorable evaluations of their relationships, especially when relationship difficulties are negotiated within broader interpersonal and normative expectations. A less self-critical and less overidentified response to relationship-related distress may be associated with lower endorsement of infidelity-related tendencies under these conditions. However, the present study did not directly assess family or marital norms, gender-role beliefs, religiosity, collectivistic orientation, perceived norms regarding infidelity, or social sanctions surrounding sexual behavior. The moderation pattern therefore should not be attributed to Turkish culture itself. Rather, the Turkish sociocultural context provides a theoretically relevant framework for interpreting the conditional association and identifies specific cultural variables that should be directly measured in future within-culture and cross-cultural research.

### 4.2 Theoretical and practical implications

The present findings make a theoretical contribution by conceptualizing self-compassion as a moderator rather than as a mediator of the association between perceived romantic relationship quality and infidelity proneness. In a cross-sectional design, a mediation model would imply a temporal process that cannot be established with the present data. The moderation framework instead asks whether the association between relationship quality and infidelity proneness differs across levels of self-compassion, which is directly consistent with the present findings.

More broadly, the results suggest that models of infidelity proneness may benefit from integrating relational context and intrapersonal characteristics rather than treating either as sufficient in isolation. Perceived romantic relationship quality showed a substantial negative bivariate association with infidelity proneness, *r* = −0.614, while self-compassion was also negatively associated with infidelity proneness, *r* = −0.251. The significant interaction further indicated that the association between perceived romantic relationship quality and infidelity proneness was not identical across individuals. In this sense, the findings support a conditional perspective in which the association between evaluations of one’s relationship and infidelity proneness partly depends on the individual characteristics brought to that relational context.

The findings may also have cautious clinical implications. Assessment of infidelity-related concerns in individual or couple therapy may benefit from considering both perceptions of relationship quality and how individuals regulate distress associated with relational difficulties. Self-compassion may be relevant to the latter process, particularly when relationship dissatisfaction is accompanied by self-criticism, shame, or emotional overidentification. However, the present findings do not establish that increasing self-compassion would reduce infidelity proneness. Given the cross-sectional design and the nonuniform interaction pattern, intervention effects should not be inferred from these data. Longitudinal and experimental research would be required before specific self-compassion-based interventions could be recommended for reducing infidelity proneness.

### 4.3 Limitations and future directions

Several limitations should be considered. First, the cross-sectional design precludes conclusions regarding temporal ordering or causality. Although perceived romantic relationship quality was specified as the focal predictor and self-compassion as the moderator for theoretical purposes, the analyses establish conditional associations rather than causal effects. Longitudinal and prospective studies are needed to determine whether relationship quality and self-compassion prospectively predict changes in infidelity proneness.

Second, all variables were assessed using self-report measures. Infidelity is a socially sensitive topic, and responses may therefore be affected by social desirability, impression management, or reluctance to disclose infidelity-related tendencies. This issue may be particularly relevant in contexts where norms surrounding sexuality, gender, and relationship exclusivity influence the acceptability of reporting such tendencies. Future studies could incorporate measures of social desirability and, where appropriate, dyadic or multimethod designs. In addition, culturally relevant factors such as beliefs concerning marriage and family cohesion, gender-role expectations, religiosity, and perceived social norms regarding infidelity were not directly assessed. Consequently, the present study cannot determine whether or how these factors shaped participants’ interpretations or reporting of relationship difficulties and infidelity proneness. Future research should directly assess these culturally relevant constructs to clarify their potential role in the associations examined here.

Third, infidelity proneness should not be equated with enacted infidelity. The present study assessed individuals’ tendency or inclination toward infidelity rather than whether infidelity subsequently occurred. This distinction is central to the present conceptualization and limits behavioral conclusions that can be drawn from the findings. Prospective research examining whether and under what circumstances infidelity proneness predicts later behavior would help clarify the behavioral significance of the construct.

Fourth, although the sample included adults across a broad age range, women constituted 70.1% of participants, and most participants were enrolled in or had completed university education. Generalizability to more gender-balanced and educationally diverse populations should therefore be examined. Participants could also be either currently involved in a romantic relationship or have been involved in one within the previous year, meaning that relationship status at the time of assessment was not uniform. Future studies could distinguish current from recently ended relationships and examine whether the moderation pattern differs by current relationship status, marital status, or relationship type.

Fifth, relationship duration was assessed categorically rather than continuously. Although dummy coding avoided treating the unequal duration categories as equally spaced, the resulting group comparisons should not be interpreted as evidence of a continuous developmental trajectory across relationship duration. Future research using a continuous measure of relationship duration could examine whether linear or nonlinear patterns better characterize its association with infidelity proneness.

Finally, the interaction accounted for a relatively modest additional proportion of variance, Δ*R*^2^ = 0.022. Although statistically reliable, the moderation finding should not be interpreted as indicating that self-compassion is a dominant determinant of the association between perceived romantic relationship quality and infidelity proneness. Replication using longitudinal data and more diverse samples is needed. Future studies could also examine other theoretically relevant moderators, including attachment insecurity, commitment, relationship-specific self-compassion, emotion regulation, and culturally shaped attitudes toward infidelity.

## 5 Conclusion

The present study suggests that perceived romantic relationship quality and self-compassion are both negatively associated with infidelity proneness and that self-compassion moderates the association between relationship quality and infidelity proneness. The negative association between perceived romantic relationship quality and infidelity proneness remained statistically significant at low, mean, and high levels of self-compassion but became progressively weaker as self-compassion increased. Rather than supporting a uniformly protective account of self-compassion, this pattern points to a more conditional role in how relational evaluations are associated with infidelity-related tendencies. By distinguishing infidelity proneness from enacted infidelity and integrating relational and intrapersonal factors, the study provides a more differentiated framework for understanding psychological vulnerability to infidelity.

## Data Availability

The raw data supporting the conclusions of this article will be made available by the authors, without undue reservation.
